# Outcome of abdominal binder in midline abdominal wound Dehiscence in terms of pain, psychological satisfaction and need for reclosure

**DOI:** 10.12669/pjms.37.4.3671

**Published:** 2021

**Authors:** Ahmed Siddique Ammar, Syed Asghar Naqi, Shehrbano Khattak, Ahmed Raza Noumani

**Affiliations:** 1 Dr. Ahmed Siddique Ammar, MBBS, MS General Surgery Senior Registrar, EAST Surgical Ward, Mayo Hospital, Lahore, Pakistan; 2 Prof. Dr. Syed Asghar Naqi, MBBS, FCPS, FRCS, MCPS-HPE Professor and Head of Surgical Department, EAST Surgical Ward, MAYO Hospital, Lahore, Pakistan. EAST Surgical Ward, Mayo Hospital, Lahore, Pakistan; 3 Dr. Shehrbano Khattak, MBBS, M-Phill Biochemistry Lecturer, Department of Biochemistry, King Edward Medical University, Lahore, Pakistan; 4 Dr. Ahmed Raza Noumani, MBBS, MS General Surgery Senior Registrar, EAST Surgical Ward, Mayo Hospital, Lahore, Pakistan

**Keywords:** Abdominal binder, burst abdomen, Dehiscence, Pain, Wound

## Abstract

**Objective:**

To assess the role of abdominal binder in patients with midline wound dehiscence after elective or emergency laparotomy in terms of pain, psychological satisfaction and need for reclosure.

**Methods:**

It was a comparative study done at EAST Surgical Ward of Mayo Hospital, Lahore from 1^st^ January 2018 to 31^st^ December 2019. One hundred and sixty-two (162) patients were included in this study with post-operative midline abdominal wound dehiscence and after informed consent by consecutive non probability sampling technique. Patients were divided into two groups by lottery method into eighty-one patients each. Group-A included patients where abdominal binder was applied and Group-B included patients without abdominal binder. In both groups pain score, psychological satisfaction and need for reclosure was assessed and compared.

**Results:**

Patients with abdominal binder shows significantly less pain (P value =0.000) and more psychological satisfaction (P value = 0.000) as compared to the patients where abdominal binder was not used. However, there was no difference in reducing the need for reclosure in patients who use abdominal binder (P value = 0.063).

**Conclusion:**

Although abdominal binder helps in reducing the pain and improving the psychological satisfaction in patients with midline abdominal wound dehiscence yet it doesn’t help in healing of wound and reclosure of the dehisced abdominal wound is needed.

## INTRODUCTION

Abdominal midline wound dehiscence or burst abdomen is very common and unusual complication of laparotomy both in elective and emergency settings. It has high morbidity especially long hospital stay as well as mortality up to 25%.^1^ Wound dehiscence or burst abdomen is defined as separation of sutured edges of the abdominal fascia after surgery.^2^ There are many factors which cause wound dehiscence like rupture of suture, knot failure, slack suture and suture cutting through the fascia but the most common cause of burst abdomen is the poor surgical technique of the surgeon closing the abdomen.^3^ Midline wound dehiscence may be partial or complete. Partial wound dehiscence may be easily overlooked and it is small separation of fascia detected only by radiological intervention. It may be ignored but it can lead to hernia formation later. Complete wound dehiscence also known as burst abdomen is clinically visible which results in secretions coming out of abdomen and in some extreme cases there is also evisceration of the abdominal contents which in turn associated with different complications like ischemia and gangrene of the intestine.^4^ The most common days of wound dehiscence is 3^rd^ to 7^th^ post-operative day but the diagnosis is often delayed especially when the skin over the rectus is also closed.^5^ Wound dehiscence is a multifactorial process which included both local and systemic factors of the body. The incidence of symptomatic wound dehiscence is about 1 % in elective surgeries. Hospital stay is also prolonged in these cases.^6^

There are many treatment options available for the management of wound dehiscence both for partial and complete. It included simple wet dressing of the wound, improve the diet of the patient and remove the factors which augment the wound dehiscence like coughing and constipation.^7^ But in complete abdominal dehiscence reclosure of the midline wound with or without retention sutures is the ultimate answer and especially in those cases where there is evisceration of the gut from the abdomen.^8^

A potential and non-pharmacological treatment option of burst abdomen is the application of abdominal binder especially during the post-operative period. Elastic binders for abdominal binder like abdominal belts, girdles trusses etc. are used in routine in different parts of the world.^9^ Abdominal binder is a wide belt which is used to hold and support the abdominal incision after surgery. There is a proven role of binder in reducing the postoperative pain, less seroma formation, better respiratory functions and postural consistency.^10^ Abdominal binders are also known to improve the mobilization, protection of the wound.so the beneficiary role of abdominal binder in postoperative recovery cannot be rejected.^11^

In this study we tried to establish tole of abdominal binder on those patients with midline abdominal dehiscence after elective or emergency surgery. To best of our literature search, we could not find studies which shows the efficacy of abdominal binder in burst abdomen.

This study will help us to understand the role of abdominal binder in patients with midline abdominal wound dehiscence as previously no literature is available to highlight the significance of role of abdominal binder once the midline laparotomy wound dehiscent occurs.

## METHODS

It was a comparative study conducted in MAYO Hospital, Lahore, which is the tertiary care hospital of Punjab and teaching hospital of King Edward Medical University (KEMU) Lahore Pakistan. The duration of the study was two years from 1^st^ January 2018 to 31^st^ December 2019. The study was approved by the Institutional Review Board of King Edward Medical University on 12/12/2017 with no 163/RC/KEMU. A total of 162 patients were selected for this study by consecutive non probability sampling technique. All those patients were selected in this study who presented in the emergency department or post-operative patients of laparotomy due to any disease admitted in ward with midline abdominal wound dehiscence. Midline abdominal wound dehiscence was defined as partial or total separation of previously approximated midline wound edges with result of sero-purulent discharge or evisceration of the gut from the midline irrespective of the technique (continuous or interrupted) of the abdominal closure used. Pain was defined by visual analogue scale of pain ranged from 0 (no pain) to 10 (worst pain). Psychological satisfaction was assessed by 5 points Likert scale ranged 1 (very unsatisfied) to 5 (very satisfied). Surgical wound dehiscence was defined as rupture or splitting open of previously closed surgical incision site.^12^

All the included patients having age more than 13 years with abdominal wound dehiscence after elective or emergency laparotomy were divided into two groups by lottery method. Group-A included those patients on which we applied the abdominal binder after wound dehiscence started and Group-B included those patients on which we didn’t applied abdominal binder after wound dehiscence and only normal saline dressings were done in these patients. Written informed consent was taken from each subject prior to the collection of data.

Abdominal binder was selected according to the abdomen diameter of the patient and was applied on the abdomen below the ribs and above the symphysis pubis after doing the normal saline dressing on the dehisced wound. Patients of both groups were allowed to open the abdominal binder while lying down but they have to close the abdominal binder before getting off the bed. Patients of both groups were allowed to had little walk with no strenuous activity and weight lifting. Factors which can aggravate the wound dehiscence like coughing constipation were treated accordingly. Patients were followed for one month after occurrence of wound dehiscence.

Data will be analyzed using SPSS version 26.0. Qualitative statistics will be determined as frequency and percentages. Quantitative correlations among variables will be determined by application of chi-square test. P Value less than 0.05 was considered significant.

## RESULTS

One hundred sixty-two patients were included in this study out of which 102 (62.2%) were male and 60 (36.6%) were female. The mean age of patients was 44 years with standard deviation of 15.28 years and minimum age of 21 years and maximum age of 85 years. Group-A patients with abdominal binder shows pain on visual analogue score of 2.45 ±1.03 while that of Group-B patients without abdominal binder shows score of 6.5 ± 1.25 (P value 0.000). Similarly, patients with abdominal binder were more satisfied psychologically with mean Likert scale of 3.97 ± 0.89 as compared to the patients without abdominal binder with mean Likert scale of 2.1 ± 0.90 (P-Values=0.000). There was no statistical difference between two group in terms of need for reclosure of the abdominal wound (56,69.1 %) in Group-A and 67,82.7% in Group-B (P-Value = 0.063) (Table-I).

**Table-I:** Comparison between patients with and without abdominal binder in terms of pain, Psychological Satisfaction and need for reclosure.

	Group-A (Patients with Abdominal binder)	Group-B (Patients without Abdominal binder)	P-Value
Pain	2.45 ± 1.03	6.5 ± 1.25	0.000
Psychological satisfaction	3.97 ± 0.89	2.1 ± 0.90	0.000
Need for reclosure	56 (69.1 %)	67 (82.7%)	0.063s
Total	81	81	162

## DISCUSSION

This study showed that wound dehiscence is significantly more in male patients (102,62.2%) as compared to female patients (60.36.6%). There was no obvious indication of the cause of this phenomenon. Some studies indicated greater physical activity contributing to the male predominance of wound dehiscence.^12^ In our study middle aged individuals (44 years) were affected more by wound dehiscence.

This study showed that the Group-A patients who used abdominal binder after wound diheascense showed less pain according to visual analogue score of 2.45 ± 1.03 as compared to the Group-B patients who didn’t used abdominal binder 6.5 ± 1.25 (P-value = 0.000). A study done by Arici et al. also showed significantly less pain score in the binder group though that study showed pain score on patients with abdominal binder but without abdominal midline wound dehiscence.^13^ Many studies also proved the role of abdominal binder in decreasing the pain after lower cesarean section.^14^ However, Gillier et al. reported no significant difference of pain by visual analogue score between binder and non-binder group.^15^ No study is yet available in literature which shows the significance of abdominal binder after midline abdominal wound dehiscence. It must be kept in mind that difference in pain levels are also related to many factors including age, gender, economic status, education level and employment etc.

The second key parameter was psychological satisfaction of the patient with abdominal wound dehiscence with abdominal binder. Our study showed that patients who used abdominal binder were satisfied and comfortable psychologically (Likert scale score of 3.97 ± 0.89) while patients who didn’t used abdominal binder were less satisfied (Likert scale score of 2.1 ± 0.90) (p-value = 0.000). This result showed that abdominal binder contributes to the psychological comfort of the patient and reduces the post-operative distress especially in the case of wound dehiscence. Rehman et al. didn’t report decreased psychological distress however that study was also not done on postoperative wound dehiscence patients.^16^ Stroker et al. showed in his review article that abdominal binder provides pain relief, improves patient satisfaction and reduces the psychologically distress post operatively.^17^

The third key parameter used was the need for reclosure of the midline abdominal wound after wound dehiscence in the presence or absence of abdominal binder. Our study showed that there was no statistical difference in both groups with and without abdominal binder (P value = 0.063). Fifty six (69.1 %) of Group-A patients with abdominal binder needs reclosure while 67 (82.7%) of patients group B who didn’t used abdominal binder needed reclosure. Indication of reclosure were evisceration of the abdominal organs, severe pain and high serous and purulent discharge from the wound.[18] This result showed that abdominal binder doesn’t help in the healing of wound after wound dehiscence and ultimately reclosure was needed.

This study will help in answering the questions regarding the effectiveness of abdominal binder in those patients with burst abdominal midline wounds. This study proved that abdominal binder has a definitive role in decreasing the pain in patients with burst abdomen and improves the psychological satisfaction in the patients with burst midline abdominal wound dehiscence.

### Limitations of the study

Limitations of the study included low sample size and single center study. More studies are needed proving the definite role of abdominal binder in wound healing after wound dehiscence.

## CONCLUSION

This study showed that abdominal binder decreases the pain and improves the psychologically satisfaction of patients with midline abdominal dehiscence after laparotomy through midline abdominal incision while abdominal binder has no role in healing of wound and ultimately reclosure in needed.

### Authors` Contribution:

**ASA and SK** conceived, designed and did statistical analysis & editing of manuscript, responsible and accountable for the accuracy or integrity of the work.

**ARN** did data collection and manuscript writing.

**SAN** review and final approval of manuscript.
